# Depressive Symptoms and the Experience of Pleasure in Daily Life: An Exploration of Associations in Early and Late Adolescence

**DOI:** 10.1007/s10802-015-0090-z

**Published:** 2015-10-26

**Authors:** Eeske van Roekel, Elise C. Bennik, Jojanneke A. Bastiaansen, Maaike Verhagen, Johan Ormel, Rutger C. M. E. Engels, Albertine J. Oldehinkel

**Affiliations:** Interdisciplinary Center Psychopathology and Emotion Regulation (ICPE), University of Groningen, University Medical Center Groningen, CC 72, P.O. Box 30001, 9700 RB Groningen, The Netherlands; Department of Education and Research, Friesland Mental Health Care Services, Leeuwarden, The Netherlands; Behavioural Science Institute, Radboud University Nijmegen, Nijmegen, The Netherlands; Trimbos Institute, Utrecht, The Netherlands

**Keywords:** Adolescence, Depressive symptoms, Anhedonia, Experience sampling method, Daily life pleasure

## Abstract

Although loss of pleasure (i.e., anhedonia) is one of the two core symptoms of depression, very little research has examined the relation between depressive symptoms and the experience of pleasure in daily life. This exploratory study in two population-based adolescent samples aimed to examine how depressive symptoms and anhedonia specifically were related to (1) the proportion and intensity of positive events, (2) mean and variability of positive affect (PA), (3) reactivity to positive events, and (4) reactivity to PA (i.e., whether PA elicits positive events). We used Experience Sampling to measure positive events and PA several times a day during 6 to 14 days in early (*N* = 284) and late (*N* = 74) adolescents. Results showed that depressive symptoms were related to a lower proportion and intensity of positive events, lower mean PA, and higher variability in PA regardless of sex and stage of adolescence. No clear evidence was found for differential reactivity to positive events or to PA. Anhedonia was not associated with most daily life experiences of pleasure. Our findings, though preliminary, suggest that although adolescents with many depressive symptoms experience less positive events and lower PA, they are able to enjoy pleasurable events to the same extent as individuals with fewer depressive symptoms.

Depression is one of the most prevalent mental disorders worldwide (World Health Organization 2012). Adolescence is a critical period in the development of mood disorders, as the prevalence of depression increases sharply in the course of adolescence (Hankin et al. 1998). Most depression research has focused on negative affect and negative experiences. This seems plausible, as according to the DSM-5 one of the two core symptoms of depression is depressed mood, and several other symptoms reflect negative emotions or cognitions (i.e., feelings of worthlessness, suicidality; American Psychiatric Association 2013). However, the other core symptom, anhedonia (i.e., loss of pleasure), is also important to investigate, because it specifically predicts worse treatment outcome (Spijker et al. 2001) and a longer and more severe course of depression (McMakin et al. 2012). Further, research showed that anhedonia experienced in adolescence is a predictor for Major Depressive Disorder (MDD) during adulthood (Wilcox and Anthony 2004).

Unfortunately, little is known about the relation between depressive symptoms and the actual experience of pleasure in daily life. Hence, it remains unclear which aspects of daily life pleasure are related to depressive symptoms. Research in adults has shown that depressive symptoms are associated with a lower frequency of positive events and lower pleasure levels during these events (i.e., lower intensity of events; Bylsma et al. 2011; Peeters et al. 2003), as well as with lower mean levels of positive affect (PA; Peeters et al. 2003; Thompson et al. 2012). In adolescents, depression has also been associated with lower mean levels of PA (Forbes et al. 2004), and with a lower positive affect/negative affect ratio (Silk et al. 2011). However, affect is not a stable entity, but fluctuates across time. Results on variability in PA in depressed adults are contradictory: depression has been related to higher general variability in PA (Gruber et al. 2013; Höhn et al. 2013; Kuppens et al. 2007), a lack of moment-to-moment variability (i.e., higher inertia) in PA (Kuppens et al. 2010), or was not associated with variability or inertia (Koval et al. 2013; Pe et al. 2014; Thompson et al. 2012). Studies in adolescents have also shown contradictory findings. Whereas one study found that emotional inertia in NA and PA during an interaction task with parents predicted the onset of MDD in adolescents 2 years later (Kuppens et al. 2012), another found that depressive symptoms in early adolescence predicted higher variability in PA, which in turn predicted more depressive symptoms 1 year later (Neumann et al. 2011). Hence, both increased and decreased variability in PA have been found to relate to depressive symptoms in adolescents. These differences in findings may be partly due to how variability was operationalized; some studies examined the standard deviation across measurements (i.e., general variability), some the auto-correlation between PA at *t* and PA at *t-1* (i.e., inertia), and some the mean squared successive difference (i.e., instability; Jahng et al. 2008; Koval et al. 2013; Thompson et al. 2012). Hence, there is a need for studies that include multiple measures of variability.

Next to the occurrence of positive events and PA as such, it is also important to investigate their interplay. Positive events are known as important factors that impact PA, but research examining whether depressive symptoms moderate the association between positive events and PA (i.e., reactivity to positive events) is lacking in adolescents. In adults, one study found evidence for a mood brightening effect, in that depressive symptoms were associated with greater increases in PA after positive events (Peeters et al. 2003). Other studies did not find differences in reactivity between depressed and non-depressed adults (Bylsma et al. 2011; Thompson et al. 2012).

The interplay between positive events and PA may also work the other way around, in that PA affects the extent to which positive events are experienced. According to positive psychology theories such as the Broaden-and-Build theory (Fredrickson 2001), positive emotions increase openness to experiences and approach behavior. This suggests that experiencing PA may elicit positive events, but no research has yet investigated this relationship in daily life, let alone the impact of depressive symptoms. As the exposure to more positive events when high in PA could further increase pleasure levels in daily life, it is important to examine whether this process is deficient in individuals suffering from depressive symptoms.

Finally, although it seems likely that the core symptom anhedonia is the driving force in the relation between depressive symptoms and the experience of pleasure in daily life, to our knowledge no studies have examined this hypothesis yet. Therefore, another aim of the present study was to examine whether anhedonia would be stronger related to the experience of pleasure in daily life than the (more heterogeneous) composite measure of depressive symptoms.

In sum, the aim of the present study was to examine relations between depressive symptoms (measured on a macro-level) and the experience of pleasure in adolescents’ daily life (measured on a micro-level). Knowledge about which of these aspects of pleasure in daily life are associated with depressive symptoms may provide important starting points for interventions. We investigated four main research questions, each representing a different aspect of daily life experiences of pleasure. First, we examined whether depressive symptoms were related to the proportion and intensity of positive events experienced in daily life (i.e., positive event characteristics). Second, the relations between depressive symptoms and mean PA and the three different types of PA variability (i.e., general variability, instability, and inertia in PA) were investigated (i.e., PA characteristics). Third, we examined whether depressive symptoms were associated with PA reactivity to positive events, and fourth, whether depressive symptoms were related to the extent to which PA elicited positive events. We hypothesized that depressive symptoms would be associated with a lower proportion and lower intensity of positive events (research question 1), and lower mean levels of PA (research question 2). With regard to the variability in PA (research question 2), we did not have a specific hypothesis, as previous research both found increased and decreased variability to be associated with depressive symptoms. Further, we expected that depressive symptoms would be associated with decreased PA reactivity to positive events (research question 3) and decreased positive event generation by PA (research question 4).

As the reward system is in full development during adolescence (Davey et al. 2008), it is possible that the association between depressive symptoms and the experience of pleasure alters during this period. Therefore, we compared an early adolescent female sample (from now on referred to as EA girls) and a late adolescent female sample (from now on referred to as LA girls) to examine age differences. Further, levels of PA and depressive symptoms differ between boys and girls (Hankin and Abramson 2001; Larson et al. 2002), which is why we compared the early adolescent female sample to an early adolescent male sample (from now on referred to as EA boys) to examine sex differences. In all three subsamples, the Experience Sampling Method (ESM) was used to measure daily life positive events and PA. The main advantages of this method are the high ecological validity and low recall bias, as participants report on their emotions and events when they experience them, in their natural environment (Myin-Germeys et al. 2009). Finally, as it seems likely that specifically the core symptom anhedonia would be associated with daily life experiences of pleasure, we examined whether the relations between depressive symptoms and the experience of pleasure in daily life are particularly driven by this symptom.

## Methods

### Participants

We used data from two different samples in the present study. Because these samples were collected for different research purposes, somewhat different inclusion and exclusion criteria were used, and some of the study procedures differed. Both samples are described in detail below.

#### Early Adolescents

The early adolescent sample was recruited through high schools in the Eastern part of The Netherlands. In schools that agreed to participate in the study (*N* = 4), all second-year students (*N* = 933) were sent an information letter to inform them about the study. When they were interested in participating, the adolescents and their parents had to sign a consent form, which was send to the principal investigator. A number of 339 adolescents returned the consent form, of which *N* = 36 could not participate in the study due to organizational issues, illness or withdrawal of consent. The resulting sample consisted of 303 adolescents (59.1 % girls), aged between 13 and 16 years (*M*_*age*_ = 14.20, *SD* = 0.54). In the present study, only adolescents who filled out at least one-third of the total number of assessments were included (i.e., more than 17 assessments), resulting in a final early adolescent sample of 284 adolescents (168 EA girls, 116 EA boys). Of this sample, 23.2 % attended preparatory secondary school for technical and vocational training, 35.9 % attended preparatory secondary school for college, and 40.5 % attended preparatory secondary school for university. The majority of adolescents were born in The Netherlands (97.2 %).

#### Late Adolescents

Dutch female late adolescents (i.e., LA girls) were recruited trough advertisements in an electronic learning environment and faculty buildings of the University of Groningen and the Hanze University of Applied Sciences. A number of 589 female students were interested in participating in the study and received an information letter. Of this group, 268 students returned the informed consent form and completed the online screening survey, which included a neuroticism questionnaire (NEO-FFI; Hoekstra et al. 1996). Based on the neuroticism scores, 50 participants were selected who scored above the 60th percentile of the sample, and 25 participants were selected who scored below the 60th percentile. This selection procedure resulted in an approximately normal distribution of neuroticism scores. In order to be included, participants had to be female, aged between 18 and 25 years, and fluent in Dutch. Exclusion criteria were inability to keep an electronic diary 5 times a day for 2 weeks, current psychiatric disorders, and standard MRI compatibility criteria. The final sample consisted of 74 females, because one participant dropped out after the first measurement day. The mean age of the final sample was 20.91 (*SD* = 1.81). Most participants attended university education (87.1 %), the other participants attended higher professional education (12.9 %).

### Procedure

#### Early Adolescents

The data collection consisted of two parts. First, participants filled out an online baseline questionnaire during school hours. Two to 8 weeks after this baseline questionnaire, and 1 day before the start of the sampling period, the procedure of the momentary assessments was explained in an individual instruction session. The sampling period comprised 6 days, during which participants were asked to fill out questionnaires 9 times per day, on a smartphone that was provided. The MyExperience program (Froehlich et al. 2007) was installed on these smartphones, which emitted buzzing signals at random time points during 90 min intervals. When adolescents received this signal, they had to fill out the questionnaire on the smartphone. When they did not respond, the buzzing signal was repeated after 2 min, with a maximum of three reminders. After this last reminder, the questionnaire was not accessible anymore. A mobile telephone number was provided, which participants could call in case of any problems. We were able to check compliance during the study, by a text message that was sent to the principal investigator whenever a questionnaire was completed. When no questionnaires were filled out within two consecutive hours, adolescents were called or sent a text message to instruct them to attend to the signals. On the final sampling day, adolescents were called to make an appointment for returning the smartphones and filling out a final short questionnaire. When adolescents completed at least 55 % of the momentary assessments, they received an incentive of €20.

#### Late Adolescents

For the ESM data collection, participants could choose whether they preferred to use (1) Personal Digital Assistants (PDAs) based on the PsyMate technology developed by Maastricht University (Myin-Germeys et al. 2009) (*N* = 33), or (2) a web-based application implemented in software for routine outcome monitoring (ROQUA, www.roqua.nl), which could be used on their own smartphone (*N* = 41).

One day before the start of the sampling period, participants were invited for an individual introduction session. During this session, they filled out baseline questionnaires and received instructions on how to use the PDA or web-based application and how to interpret the questions. The sampling period consisted of 14 days, during which participants filled out five questionnaires per day, at fixed time-points with 3-h intervals. The time points were adapted to an individuals’ daily rhythm. Programmed auditory signals (PDA’s) or web-based generated text messages (web-based application) informed participants that they had to fill out a new questionnaire. Participants were instructed to complete the questionnaire as soon as possible, at least within 30 min after the signal. Contact information of the researchers was available in case of any problems. Participants were contacted at the end of the first week to check the progress. All participants received €30 for participation. Those who completed at least 63 out of 70 measurements received an additional bonus of €20.

### Materials

#### Depressive Symptoms

Depressive symptoms were measured with the Symptom Checklist (SCL-90; Derogatis and Cleary 1977) in the LA subsample and with the Brief Symptom Inventory (BSI, i.e., the brief SCL-90, adapted for adolescents; Derogatis and Melisaratos 1983) in the EA subsample. As the depressive symptoms subscale of the SCL-90 included more items than the subscale of the BSI, we selected only those items that were included in both scales. Some items were phrased slightly different for adolescents, but the meaning of these items was similar. For both scales, participants rated the extent to which they experienced the following items on a 5-point scale (range = 1–5): “thoughts of ending your life”, “feeling lonely”, “feeling blue”, “feeling no interest in things”, “feeling hopeless about the future”, “feelings of worthlessness”. Higher scores on this scale were indicative of more depressive symptoms.

#### Anhedonia

The item we used to measure anhedonia was part of the depressive symptoms scale and read “feeling no interest in things”. A higher score on this item was indicative of higher levels of anhedonia (range = 1–5).

#### Positive Affect (PA)

PA was measured in all subsamples at each momentary assessment with four items: I feel cheerful, relaxed, content, and energetic, which were rated on a 7-point scale (range = 1–7). Cronbach’s alpha was 0.77 for EA girls, 0.74 in EA boys, and 0.80 for LA girls (calculated over all assessments). Higher scores were indicative of higher levels of PA. The variability in PA was measured in three ways. For general variability, we calculated the standard deviation of PA over all momentary assessments within one individual. For instability, we calculated the mean squared successive difference (MSSD) within individuals. This is done by subtracting subsequent PA scores from each other (i.e., difference score), and squaring these scores. To avoid evening to morning differences, we excluded difference scores that represented mood changes between days. Subsequently, the mean square difference score is calculated by summing all squared difference scores and dividing them between the total number of assessments minus 1 (i.e., the number of difference scores). Finally, we took the square root of the MSSD to correct for the positive skewness (i.e., RMSSD). The RMSSD is suggested to be a preferred index for instability, because it has features of both variability and temporal dependency (Jahng et al. 2008). For both the mean SD and the RMSSD, higher scores were indicative of higher levels of variability. For inertia, we analyzed the auto-correlation between PA at *T-1* and PA at *T.*

#### Positive Events

At each assessment, participants were asked to report what the most important event was since the last assessment. This event was subsequently rated on pleasantness on a 7-point scale (−3 = *very unpleasant*, 0 = *neutral*, 3 = *very pleasant*). As we focused on positive events only, we recoded the negative scores (i.e., −3, −2, and −1) into 0. Hence, a higher score on this variable reflected a more pleasurable event (range = 0–3). To calculate the proportion of positive events, we counted the number of positive events per individual, and divided this by the total number of assessments for that individual. Hence, a score close to zero on this variable indicated almost no reports of positive events, a score close to 1 indicated that nearly all events flagged as most important were positive.

#### Time Lags

As the time between assessments differed between the EA and LA subsamples, we included only those assessments that were filled out between 30 and 240 min after the previous assessment in the analyses that included lagged variables (i.e., variables measured at the previous assessment).

### Strategy of Analyses

Descriptive statistics were calculated, and differences between subsamples in mean levels of depressive feelings, anhedonia, PA, moment-to-moment variability (i.e., RMSSD), general variability (mean SD), and the proportion of positive events were tested by conducting ANOVAs.

Subsequently, associations between depressive symptoms and positive event characteristics were examined. Associations between depressive symptoms and the proportion of positive events were explored by using regression analyses in SPSS, whereas the association between depressive symptoms and the intensity of positive events was examined by using multilevel analyses in Mplus, as the multiple assessments are nested within individuals (i.e., Level 1 = assessment level, Level 2 = individual level). Multilevel analyses enable the examination of relations between Level 1 (e.g., positive events; dependent variable) and Level 2 variables (e.g., depressive symptoms; independent variable).

Next, the associations between depressive symptoms and PA characteristics were investigated. The relation between depressive symptoms and the mean level of PA was examined in a multilevel analysis with PA as dependent variable and depressive symptoms as independent variable. Inertia (i.e., instability in PA) was examined in a multilevel model in which PA_*T-1*_ and PA_*T*_ (i.e., the autocorrelation of PA) were included as independent and dependent variable, respectively. We examined whether depressive symptoms affected inertia by including the cross-level interaction between depressive symptoms and PA_*T-1*_. The relation between depressive symptoms and general variability of positive affect and general instability was examined by two regression analyses with the SD and the RMSSD as dependent variables, respectively, and depressive symptoms as independent variables.

The relation between positive events and PA (i.e., PA reactivity to positive events) was examined in a multilevel model in which positive events was included as independent variable and PA_*T*_ as dependent variable, while controlling for PA_*T-1*_. Moderation of depressive symptoms was examined by adding the cross-level interaction with positive events.

We investigated the relation between PA_*T-1*_ and positive events at *T* (i.e., positive event generation by PA), and whether depressive symptoms moderated this relation, again in a multilevel model, with PA_*T-1*_ and the cross-level interaction between PA_*T-1*_ and depressive symptoms as independent variables and positive events at *T* as dependent variable, while controlling for positive events at *T-1*. For all analyses described above, we replaced depressive symptoms with anhedonia to examine whether the found associations were driven by anhedonia.

For all models, we examined whether the relations differed between EA girls versus EA boys (i.e., sex differences), and between EA girls versus LA girls (i.e., age differences). This was done by including dummy variables for subsample and subsample by depressive symptoms interactions in the regression analyses, and by conducting multi-group analyses in the multilevel models. In the multi-group analyses we examined whether the model fit (*∆χ*^2^) of the multilevel model in which the paths of interest are allowed to differ between subsamples was significantly better than the model fit of the model in which all paths were constrained to be equal across subsamples.

Predictors at the individual level (i.e., depressive symptoms and anhedonia) were standardized within subsamples and predictors at the assessment level (i.e., PA, positive events) were centered within individuals (i.e., person-specific mean centering). Significance levels (two-tailed) were set at *p* < 0.05 for all models. Missing data were not imputed; the multilevel models used all data that were available for each adolescent. That is, for some adolescents 20 assessments were included in the analyses, whereas for others 54 assessments were included. For multilevel analyses, the recommended sample size is a minimum of 20 observations (momentary assessments) for at least 50 groups (individuals) (Maas and Hox 2005). Our smallest subgroup consisted of 74 participants, showing that we had enough power for our analyses.

## Results

### Descriptive Statistics

Table 1 shows the mean levels of the model variables in the different samples. EA girls reported significantly more depressive symptoms and anhedonia than LA girls. Scores on depressive symptoms (means =1.36–1.60; 75th percentile EA girls =1.83, EA boys =1.67, LA girls =1.50) and anhedonia (means =1.12–1.92; 75th percentile EA girls =2.00, EA boys = 2.00, LA girls =1.00) were relatively low, given the range (range = 1–5), whereas mean levels of PA (means =4.57–5.05) were relatively high (range = 1–7). Mean levels of PA were higher in EA girls, compared to LA girls, whereas the proportion of positive events was higher in LA girls compared to EA girls. General variability and general instability were higher in EA girls than in LA girls. EA boys had similar levels of mean PA, general instability, and a similar proportion of positive events as EA girls. General variability and depressive symptoms were higher in EA girls than in EA boys. As can be seen in Table 2, the variability measures were correlated in all samples. Further, higher mean levels of PA were related to lower variability in PA.

**Table 1:** Descriptive statistics for each sample

	EA boys			EA girls			LA girls				
	M (SD)	Range	N	M (SD)	Range	N	M (SD)	Range	N	F	df
Age	14.25^a^ (0.54)	13–16	116	14.15^a^ (0.54)	13–16	168	20.91^b^ (1.81)	18–25	74	1416.87***	357
Depressive symptoms	1.43^a^ (0.44)	1.00–3.33	116	1.60^b^ (0.62)	1.00–4.67	163	1.36^a^ (0.46)	1.00–3.50	74	6.29**	352
Anhedonia	1.92^a^ (0.79)	1.00–5.00	116	1.74^a^ (0.87)	1.00–5.00	163	1.12^b^ (0.50)	1.00–4.00	74	25.01***	352
Mean PA	5.05^a^ (0.76)	2.87–6.70	116	4.97^a^ (0.72)	2.98–6.41	168	4.57^b^ (0.67)	2.59–5.96	74	10.72***	357
RMSSD	0.99^ab^ (0.39)	0.25–2.21	116	1.08^a^ (0.36)	0.41–2.15	168	0.90^b^ (0.25)	0.42–1.67	74	7.57**	357
Mean SD PA	0.82^a^ (0.29)	0.24–1.71	116	0.91^b^ (0.28)	0.29–1.95	168	0.77^a^ (0.21)	0.31–1.47	74	7.05***	357
Proportion PE	0.56^a^ (0.19)	0.05–0.97	116	0.56^a^ (0.17)	0.07–0.95	168	0.62^b^ (0.20)	0.00–0.96	74	3.31*	357
Intensity PE	1.33^a^ (0.52)	0.10–2.45	116	1.33^a^ (0.46)	0.21–2.53	168	1.01^b^ (0.38)	0.00–2.09	74	13.57***	357

Means are compared horizontally. Mean levels with similar superscripts do not significantly differ from each other (e.g., both ^a^). Mean levels with different superscripts significantly differ from each other (e.g., ^a^, ^b^, and ^c^). *PA* positive affect, *PE* positive events, *RMSSD* root mean squared successive difference, *SD* standard deviation, *EA* early adolescent, *LA* late adolescent. *p < 0.05. **p < 0.01. ***p < 0.001.

**Table 2:** Correlations between model variables, split per sample

	EA boys						EA girls						LA girls					
	1.	2.	3.	4.	5.	6.	1.	2.	3.	4.	5.	6.	1.	2.	3.	4.	5.	6.
1. Depressive symptoms	–						–						–					
2. Anhedonia	0.43***	–					0.57***	–					0.73***	–				
3. Mean PA	−0.36***	−0.12	–				−0.22**	0.01	–				−0.39**	−0.30*	–			
4. RMSSD	0.20*	0.12	−0.44***	–			0.10	0.03	−0.37***	–			0.10	−0.05	−0.44***	–		
5. Mean SD	0.24**	0.15	−0.42***	0.86***	–		0.11	0.08	−0.41***	0.85***	–		0.14	−0.05	−0.47***	0.89***	–	
6. Proportion positive events	−0.23*	−0.21*	0.22*	−0.14	−0.11	–	−0.13	0.05	0.28**	−0.18*	−0.24**		−0.31**	−0.31**	0.49***	−0.11	−0.17	–
7. Intensity positive events	−0.28**	−0.16	0.29**	0.03	0.06	0.88***	−0.09	0.10	0.36***	−0.06	−0.12	0.88***	−0.26*	−0.28*	0.51***	−0.07	−0.09	0.86***

*PA* Positive affect, *RMSSD* Root mean squared successive difference, *SD* standard deviation, *EA* early adolescent, *LA* late adolescent. *p < 0.05. **p < 0.01. ***p < 0.001.

### Depressive Symptoms and Positive Event Characteristics

Results showed that depressive symptoms were related to a lower proportion of positive events (*B* = −0.04, *SE* = 0.01, *p* < 0.001). Depressive symptoms were also related to less pleasure during positive events (*B* = −0.09, *SE* = 0.03, *p* < 0.01). Anhedonia was negatively related to the proportion of positive events (*B* = −0.02, *SE* = 0.01, *p* < 0.05), but not related to the level of pleasure during events (*B* = −0.03, *SE* = 0.03, *p* = 0.30).

### Depressive Symptoms and PA Characteristics

Depressive symptoms were related to lower levels of PA (*B* = −0.22, *SE* = 0.04, *p* < 0.001) Depressive symptoms were related to higher levels of general variability (i.e., SD; *B* = 0.04, *SE* = 0.01, *p* < 0.01) and higher levels of instability (i.e., RMSSD; *B* = 0.05, *SE* = 0.03, *p* < 0.01). The auto-correlation between PA_*T-1*_ and PA_*T*_ was significant (*B* = 0.27, *SE* = 0.02, *p* < 0.001). Depressive symptoms did not affect the autocorrelation between PA_*T-1*_ and PA_*T*_ (*B* = −0.00, *SE* = 0.01, *p* = 0.80), indicating no association between depressive symptoms and inertia in PA.

Anhedonia was not related to PA (*B* = −0.07, *SE* = 0.05, *p* = 0.13), nor general variability in PA (*B*_*SD*_ = 0.02, *SE* = 0.02, *p* = 0.16) nor instability (*B*_*RMSSD*_ = 0.02, *SE* = 0.02, *p* = 0.38), and did not affect the autocorrelation between PA_*T-1*_ and PA_*T*_ (*B* = 0.00, *SE* = 0.01, *p* = 0.81).

### Depressive Symptoms and PA Reactivity to Positive Events

Positive events were related to PA (*B* = 0.15, *SE* = 0.01, *p* < 0.001). The cross-level interaction between depressive symptoms and positive events on PA was borderline significant (*B* = 0.02, *SE* = 0.01, *p* = 0.05). This finding tentatively indicates that adolescents with many depressive symptoms increased more in positive affect when they experienced more positive events. Anhedonia did not moderate the relation between positive events and PA (*B* = 0.01, *SE* = 0.01, *p* = 0.34).

### Depressive Symptoms and Positive Event Generation by PA

PA_T-1_ was positively related to positive events at *T* (*B* = 0.04, *SE* = 0.01, *p* < 0.01). Depressive symptoms did not moderate the relation between PA_T-1_ and positive events (*B* = 0.01, *SE* = 0.01, *p* = 0.48). Anhedonia did not moderate the relation between PA_*T-1*_ and positive events at *T* (*B* = 0.00, *SE* = 0.01, *p* = 0.89).

### Age and Sex Differences

#### Positive Event Characteristics

**Fig. 1:**
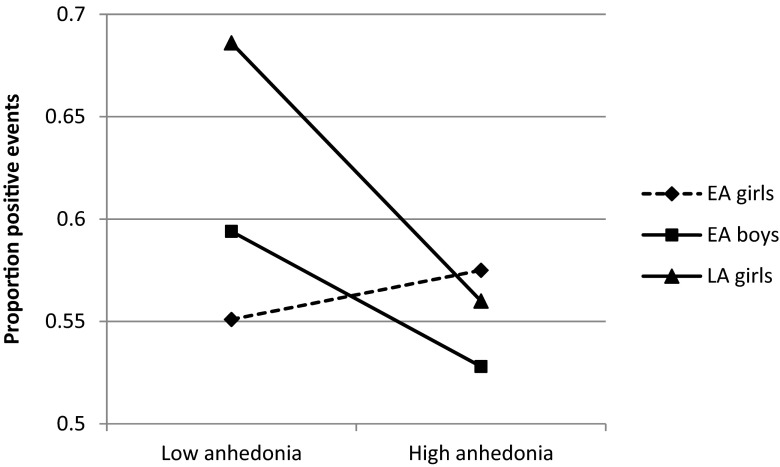
Relation between anhedonia and proportion of positive events, split per sample. *Note*. Low anhedonia reflects M – 1SD, high anhedonia reflects M + 1SD. The *solid lines* represent significant slopes, the *dashed line* represents a non-significant slope

For the aggregated variables, differences between subsamples were tested by means of interactions. No age and sex differences were found for the relation between depressive symptoms and the proportion of positive events (*B* = −0.02, *SE* = 0.02, *p* = 0.33 for EA boys versus EA girls; *B* = −0.04, *SE* = 0.03, *p* = 0.11 for LA girls versus EA girls). With regard to the relation between anhedonia and the proportion of positive events, significant subsample differences were found (*B* = −0.05, *SE* = 0.02, *p* < 0.05 for EA boys versus EA girls; *B* = −0.07, *SE* = 0.03, *p* < 0.05 for LA girls versus EA girls). As can be seen in Fig. 1, anhedonia was not related to the proportion of positive events in EA girls, whereas in EA boys and LA girls, higher levels of anhedonia predicted a lower proportion of positive events. Further, as can be seen in Table 3, no differences were found for the association between depressive symptoms and the intensity of positive events (model 1a). We did find significant differences between subsamples in the relation between anhedonia and the intensity of positive events. Significant age differences were present, in that for LA girls, higher levels of anhedonia were related to lower levels of pleasure during events, whereas for EA girls this relation was not significant. No significant sex differences were found between EA girls and EA boys (LA girls: *B* = −0.11, *SE* = 0.05, *p* < 0.05; EA girls: *B* = 0.05, *SE* = 0.03, *p* = 0.13; EA boys: *B* = −0.08, *SE* = 0.07, *p* = 0.21).

**Table 3:** Results for chi square difference tests

	Total sample			EA girls versus EA boys			EA girls versus LA girls		
	*Δχ* ^2^	*df*	*p*	*Δχ* ^2^	*df*	*p*	*Δχ* ^2^	*df*	*p*
1. a. DS on intensity positive events	3.18	2	0.20						
1. b. ANH on intensity positive events	8.11	2	< 0.05	2.75	1	0.10	54.45	1	< 0.001
2. a. DS on PA	1.91	2	0.38						
2. b. ANH on PA	5.29	2	0.07						
2. c. PA (T-1) on PA (T)	5.71	2	0.06						
2. d. Moderation DS	0.46	2	0.79						
2. e. Moderation ANH	0.33	2	0.84						
3. a. Positive events (T) on PA (T)	160.43	2	< 0.001	3.23	1	0.07	57.30	1	< 0.001
3. b. Moderation DS	1.15	2	0.56						
3. c. Moderation ANH	0.37	2	0.83						
4. a. PA (T-1) on positive events (T)	31.95	2	< 0.001	3.25	1	0.07	15.50	1	< 0.001
4. b. Moderation DS	0.05	2	0.80						
4. c. Moderation ANH	0.03	2	0.99						

*DS* Depressive symptoms, *ANH* anhedonia, *PA* positive affect, *EA* early adolescent, *LA* late adolescent

#### PA Characteristics

No significant differences were found between subsamples in the relation between depressive symptoms and the variability measures (*B*_*SD*_ = 0.04, *SE* = 0.03, *p* = 0.22 and *B*_*RMSSD*_ = 0.04, *SE* = 0.04, *p* = 0.33 for EA boys versus EA girls; *B*_*SD*_ = −0.00, *SE* = 0.04, *p* = 0.98 and *B*_*RMSSD*_ = −0.01, *SE* = 0.01, *p* = 0.82 for LA girls versus EA girls) nor for anhedonia (*B*_*SD*_ = 0.02, *SE* = 0.03, *p* = 0.52 and *B*_*RMSSD*_ = 0.04, *SE* = 0.05, *p* = 0.41 for EA girls versus EA boys; *B*_*SD*_ = −0.03, *SE* = 0.04, *p* = 0.38 and *B*_*RMSSD*_ = −0.02, *SE* = 0.05, *p* = 0.63 for EA girls versus LA girls). As can be seen in Table 3, no subsample differences emerged in the autocorrelation of PA (model 2c) nor in the effect of depressive symptoms and anhedonia on the autocorrelation (models 2d and 2e).

#### PA Reactivity to Positive Events

Significant differences were found between the subsamples in the relation between positive events and PA (model 3a in Table 3), in that this relation was stronger for LA girls, compared to EA girls (LA girls: *B* = 0.32, *SE* = 0.02, *p* < 0.001; EA girls *B* = 0.11, *SE* = 0.01, *p* < 0.001). EA girls did not differ from EA boys (EA boys: *B* = 0.08, *SE* = 0.02, *p* < 0.001). No subsample differences were found for moderation with depressive symptoms (model 3b) and anhedonia (model 3c) in this association.

#### Positive Event Generation by PA

Significant differences were found between subsamples in the relation between PA_*T-1*_ and positive events at *T*. In EA girls and EA boys, the relation between PA_*T-1*_ and positive events at *T* was not significant (EA girls: *B* = 0.03, *SE* = 0.02, *p* = 0.23; EA boys: *B* = −0.03, *SE* = 0.03, *p* = 0.31, respectively), whereas in LA girls high levels of PA at the previous assessment predicted more pleasure during events at the current assessment (*B* = 0.08, *SE* = 0.02, *p* < 0.001). Again, the moderation by depressive symptoms (model 4b) and anhedonia (model 4c) in this association did not differ between the subsamples.

## Discussion

This study aimed to examine relations between depressive symptoms and experience of pleasure in daily life in early and late adolescents. Results showed that depressive symptoms were related to a lower mean PA, lower proportion of positive events, lower intensity of positive events, and higher general variability in PA regardless of sex and stage of adolescence. We found evidence for reactivity to positive events and to PA (i.e. whether PA elicits positive events), which were both strongest in LA girls. However, no clear evidence was found for moderation of these associations by depressive symptoms. Anhedonia did not drive the associations between depressive symptoms and momentary experiences of pleasure, as it was only related to the proportion of positive events.

Our findings indicated that individuals with many depressive symptoms experienced fewer positive events and less pleasure during these events, which is in line with previous research in adults (Bylsma et al. 2011; Peeters et al. 2003; Thompson et al. 2012). However, we did not find robust evidence that individuals with many depressive symptoms differed in PA reactivity to positive events. Hence, even though they experienced less pleasure and PA, their PA levels increased after positive events to the same extent as individuals with fewer depressive symptoms. We even found a slight indication for the mood brightening effect, which has been previously reported in adults (Bylsma et al. 2011; Peeters et al. 2003; Thompson et al. 2012). These findings indicate that the ability to increase PA after positive events is not affected by depressive symptoms.

With regard to the variability in PA, we found that depressive symptoms were associated with higher general variability and instability (i.e., greater SD and MSSD of PA), but not with lower inertia of PA. It may seem contradictive that we did find an association with the MSSD but not with the autocorrelation of PA, as they both relate to the frequency of shifts. The main difference between these two measures is that the autocorrelation takes the mean level of PA into account, and the MSSD does not. Hence, a strong negative autocorrelation merely reflects frequent shifts around the mean level of PA and a high MSSD indicates frequent and large shifts in PA, irrespective of the mean level of PA. The finding that inertia in PA was not associated with depressive symptoms thus indicates that depressive symptoms are not associated with more fluctuations around the mean level of PA, which is measured by the autocorrelation. Rather, depressive symptoms are related to a greater range in PA levels. These diverging findings illustrate the importance of taking into account multiple measures of variability in one study.

As we found little evidence for differential reactivity to positive events in individuals with many depressive symptoms, it seems implausible that the increased variability in PA in individuals with many depressive symptoms is caused by different responses to positive events. Since it has been shown that adolescents at risk for psychopathology respond more negatively to negative events (Schneiders et al. 2006) and that adolescents with many depressive symptoms benefit more from being with close others (Brown et al. 2011), depression-related shifts in PA are more likely due to differential reactivity to negative events or to enduring characteristics of the social context.

Our daily life measures of PA and positive events may reflect two different types of anhedonic processes. The level of PA during and after positive events may reflect consummatory anhedonia, that is, the ability to enjoy a positive event when it happens (Treadway and Zald 2011). The proportion of positive events may be more strongly related to motivational anhedonia, which is a lack of motivation or desire to engage in pleasurable activities. From this perspective, our findings are inconsistent regarding consummatory anhedonia: Depressive symptoms were related to a lower level of pleasure during positive events (i.e., intensity of positive events), but not associated with the experience of PA in response to positive events. Hence, although adolescents with many depressive symptoms experienced lower levels of pleasure during positive events, they responded similarly to these events as their levels of PA increased to the same extent as in adolescents with fewer depressive symptoms. A possible explanation for these differences in findings with regard to consummatory anhedonia may lie in how we measured these two concepts. The intensity of events was asked directly, as participants rated how pleasurable the most important event was. Therefore, the negative bias that is associated with depressive symptoms may be activated here. For the reactivity to events, we created a link between events and PA in the analyses, hence participants themselves did not consciously make a link between the events and how they felt. The negative bias may thus play less of a role in the assessment of reactivity to events. Further research could try to overcome these issues by including a more objective measure of events (i.e., by having participants describe the most important event, and have these answers rated on level of pleasure by independent coders).

Motivational anhedonia may explain why we found that individuals with many depressive symptoms did not experience as many positive events as individuals with fewer depressive symptoms, as they may not go out and participate in pleasurable activities. To investigate the role of motivational anhedonia, further research should include questions about momentary motivation or drive.

Both age and sex differences were found in the experience of pleasure in daily life. First, EA girls showed greater variability in PA than LA girls and EA boys, which may indicate that there are sex differences in PA variability in early adolescents, and that affect stabilizes in the course of adolescence. Other studies have also found that girls are more variable in PA than boys (Larson and Csikszentmihalyi 1980) and that affect stabilizes throughout adolescence, in both boys and girls (Larson et al. 2002).

Second, the relation between positive events and PA was stronger in LA girls than in EA girls. A possible explanation for this finding may be that the timeframe between two assessments was different between the two samples. Although the amount of time between assessments did not affect the results we found when we controlled for it, it could be that the events reported by the LA sample may have been more important, because the timeframe was larger and hence there were more opportunities for greater events to happen.

Third, PA at a certain time point elicited more positive events at the next time point in LA girls, but not in EA girls and EA boys. A developmental explanation for this age difference could be that young adolescents do not yet have the capacity to elicit more positive events when they are high in PA. To our knowledge, no studies have yet examined age differences in how high levels of PA affect individuals. Further, the EA samples could have had less freedom to choose the activities they wanted to do, because they spent a great amount of time at school and at home with their parents. The older sample consisted mainly of college students who lived on their own (i.e., > 95 %) and did not spend as much time at school as the younger subsamples. Hence, the younger samples may have had the same capacity to experience positive events in case of high PA as the older one, but not be able to materialize this capacity due to their more rigid schedule.

Finally, our findings seem to indicate that anhedonia is more important for the experience of pleasure in daily life later than early in adolescence, as we found that anhedonia was related to the proportion of positive events or pleasure levels during events in LA girls only. Further research is needed to examine the development of anhedonia throughout adolescence.

### Limitations

Our study has several limitations. First, although we could examine sex and age differences, we did not have a sample of older boys, and hence could not investigate sex differences in late adolescence. Further research is needed to examine whether some of the differences between EA girls and LA girls are similar for boys. Related to this, we did not measure pubertal development in the early adolescent sample. Previous research has shown that pubertal phase is associated with altered brain reactivity to rewards, with adolescents in mid-late puberty showing increased striatal reactivity to rewards (Forbes et al. 2010). Hence, puberty may affect daily life pleasure within the early adolescent sample. Although it is likely that most adolescents in our sample were in the mid-late pubertal phase, given their age (mean age = 14.20), it would have been interesting to examine whether pubertal stage could explain some of the sex and age differences we found. Second, our samples consisted of healthy individuals and therefore, the mean scores of depressive symptoms were relatively low. To assess potential treatment implications, it is important to examine to what extent the associations found in our samples can be generalized to adolescent patient samples.

A third limitation concerns the depression and anhedonia measures used in this study. Our depression measure only included cognitive-affective symptoms and no somatic symptoms (e.g., fatigue, appetite, sleep abnormalities). Cognitive-affective symptoms are expected to be more strongly related to daily life experiences of pleasure than somatic symptoms, but this is an untested assumption. Moreover, a more extensive measure of anhedonia is warranted. Our 1-item measure that focused on loss of interest (i.e., motivational aspect of pleasure) was related only to the proportion of positive events, which also reflects motivational aspects of anhedonia rather than consummatory aspects. Possibly, an anhedonia measure that differentiates between aspects such as consummatory and motivational anhedonia will reveal more specific associations with daily life experiences of pleasure. The difference in findings between anhedonia and depressive symptoms may be explained by the fact that the anhedonia measure included only one item, whereas the depressive symptoms measure consisted of 6 items. Therefore, the latter measure may be better in explaining daily life experiences, as it is likely a more reliable measure with a greater range.

Fourth, although we used very similar measures in both samples, there were some differences in design that may have affected the results. The main difference was that the time between assessments was larger in the LA subsample. Controlling for the time between assessments did not affect our findings directly, yet it may have impacted the importance of the reported events. Related to this, the amount of time between the assessment of depressive symptoms and the ESM procedure varied between samples. In LA girls, the baseline was consistently administered 1 day prior to the ESM. In EA boys and girls, the baseline questionnaire was administered 2 to 8 weeks prior to the ESM and so may have changed in the period in between. However, our results do not consistently show stronger relations between depressive symptoms and ESM measures in LA girls, which suggests that this time difference has not affected our results to a great extent.

Finally, there are many other factors that could influence daily life pleasure that were not included in the present study. For example, previous research using fMRI has shown that sleep deprivation (Holm et al. 2009; Mullin et al. 2013), peer victimization, and low parental warmth (Casement et al. 2014) are associated with reduced reward reactivity. Hence, further research could examine whether these factors affect or explain the association between depressive symptoms and daily life experiences of pleasure.

## Conclusion

In conclusion, this exploratory study showed that depressive symptoms were associated with a lower proportion of positive events and less experienced pleasure during these events in both early and late adolescents. Nevertheless, individuals with many depressive symptoms equally benefited from positive events as adolescents with fewer depressive symptoms, showing that the capacity to enjoy pleasurable events is not affected. These associations were not primarily driven by anhedonia, as anhedonia had little effect on the experience of pleasure in daily life. Pending future research on this topic, interventions might focus on stimulating positive events in individuals with many depressive symptoms, possibly by increasing the motivation to engage in pleasurable activities.
